# Disseminated *Mycobacterium chelonae* infection predominantly involving the facial region of an immunocompromised elderly patient

**DOI:** 10.1093/qjmed/hcaf176

**Published:** 2025-08-01

**Authors:** Yosuke Sazumi, Hideharu Hagiya, Shinnosuke Fukushima, Jumpei Uchiyama, Poowadon Muenraya, Satoru Sugihara, Yoshio Kawakami, Shin Morizane, Kohei Oguni, Fumio Otsuka

**Affiliations:** Department of General Medicine, Okayama University Hospital, Okayama, Japan; Department of Infectious Diseases, Okayama University Hospital, Okayama, Japan; Department of Infectious Diseases, Okayama University Hospital, Okayama, Japan; Department of Bacteriology, Graduate School of Medicine Dentistry and Pharmaceutical Sciences, Okayama University, Okayama, Japan; Department of Bacteriology, Graduate School of Medicine Dentistry and Pharmaceutical Sciences, Okayama University, Okayama, Japan; Department of Dermatology, Okayama University Hospital, Okayama, Japan; Department of Dermatology, Okayama University Hospital, Okayama, Japan; Department of Dermatology, Okayama University Hospital, Okayama, Japan; Department of General Medicine, Okayama University Hospital, Okayama, Japan; Department of Infectious Diseases, Okayama University Hospital, Okayama, Japan; Department of General Medicine, Okayama University Hospital, Okayama, Japan; Department of Infectious Diseases, Okayama University Hospital, Okayama, Japan

**Figure 1.**
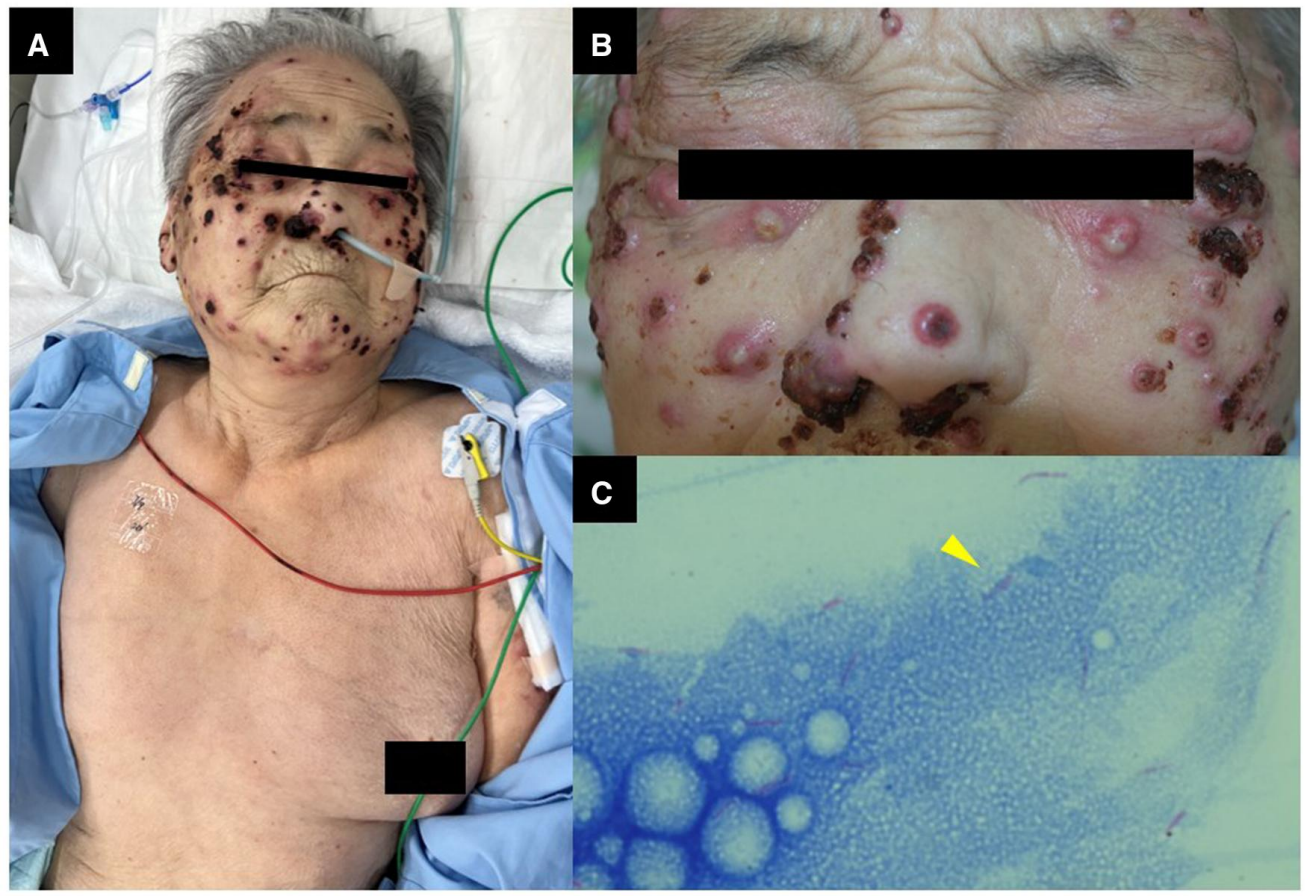
Dermatological manifestations and microbiological findings. (**A**, **B**) Multiple erythematous pustular lesions are evident on the facial region, with predominant distribution across the malar and nasal surfaces, accompanied by focal ulcerative changes. The cutaneous examination revealed an absence of such dermatological manifestations on her trunk. (**C**) Ziehl-Neelsen acid-fast staining of the pustular biopsy specimen demonstrated abundant acid-fast bacilli, consistent with mycobacterial infection.

An 85-year-old woman with a preceding history of polymyalgia rheumatica, for which oral corticosteroids and tacrolimus had been prescribed for a 5-year duration, was transferred to our hospital with a chief complaint of numerous pustular lesions. The dermatological manifestations were distributed predominantly across her face and upper extremities, whereas the truncal area remained unaffected by such cutaneous lesions (Figure 1A and B). The painful skin eruption initially emerged ∼3 months earlier on her right lower limb, with several lesions subsequently progressing to ulcerative stages. At another hospital, although bacterial culture of a skin biopsy specimen yielded unremarkable findings, histopathological examination revealed significant neutrophilic infiltration within the dermis and subcutaneous adipose tissue, resulting in a clinical diagnosis of pyoderma gangrenosum.

After admission to our hospital, the patient underwent a multiple skin biopsy of the pustular lesions, wherein acid-fast staining revealed evidence of mycobacterial infection (Figure 1C). Polymerase chain reaction testing for *Mycobacterium tuberculosis* was negative, while mycobacterial culture identified a rapidly growing mycobacterial species, which was later determined to be *Mycobacterium chelonae* by mass spectrometry (MALDI Biotyper; Bruker Daltonics, Billerica, MA, USA). Further short-lead genome analysis corroborated the organism to be *M.chelonae*. The raw sequencing data were deposited to DDBJ Read Archive (accession No. DRR701379). The pathogen was also isolated from blood culture, and radiologic investigations showed multiple pustular lesions on the face and limbs, leading to a final diagnosis of disseminated *M.chelonae* infection. Antimicrobial combination therapy with tobramycin, imipenem/cilastatin and clarithromycin was initiated; however, the patient developed septic shock and expired on the 14th day of hospitalization.

*Mycobacterium chelonae* is one of the representative rapidly growing nontuberculous mycobacteria that is widely distributed in the environmental reservoirs, particularly characterized by an optimal growth temperature of 28–30°C.^1^ This ubiquitous pathogen potentially causes skin and soft tissue infections in immunocompromised patients in a chronic manner, with multiple painful, erythematous nodules with purulent drainage.^2^ Because of the lower temperature, cutaneous *M.chelonae* infections most commonly affect the distal extremities in human hosts.^3^ Notably, the cutaneous lesions in the present patient were exclusively localized to the facial region, as well as the limbs. This phenomenon can be explained by the fact that facial skin temperature, particularly in the nasal, malar and glabellar areas, is known to decrease with advancing age.^4^ In this case, the prolonged history of steroid-induced diabetes mellitus may have contributed to autonomic dysfunction, further lowering the facial skin temperature. This case highlighted that nontuberculous mycobacteria infection should be considered in the differential diagnosis of patients presenting with multiple pustular lesions on exposed areas.
